# Research progress on the role of adipocyte exosomes in cancer progression

**DOI:** 10.32604/or.2024.043482

**Published:** 2024-09-18

**Authors:** YUN WANG, XIAOJIANG LI, DALONG LIU, ZHIFENG WANG, JICHEN XIA, LIJUN WANG, XUDONG ZHANG

**Affiliations:** 1 Department of Breast and Thyroid Surgery, Affiliated Hospital of Changchun University of Traditional Chinese Medicine, Changchun, 130021, China; 2 Department of Orthopaedics, Affiliated Hospital of Changchun University of Traditional Chinese Medicine, Changchun, 130021, China; 3 Department of Internal Medicine, Changchun Chaoyang District Hospital of Traditional Chinese Medicine, Changchun, 130061, China; 4 Department of Orthopedics and Traumatology, Jilin Integrated Traditional Chinese and Western Medicine Hospital of Jilin Province, Jilin, 132012, China; 5 Department of Oncology, Liaoyuan Second People’s Hospital, Liaoyuan, 136299, China; 6 Department of Brain Surgery, Affiliated Hospital of Changchun University of Traditional Chinese Medicine, Changchun, 130021, China

**Keywords:** Exosomes, Extracellular vesicles, Adipose tissue, Tumor microenvironment, Obesity

## Abstract

Exosomes, minute vesicles ubiquitously released by diverse cell types, serve as critical mediators in intercellular communication. Their pathophysiological relevance, especially in malignancies, has garnered significant attention. A meticulous exploration of the exosomal impact on cancer development has unveiled avenues for innovative and clinically valuable techniques. The cargo conveyed by exosomes exerts transformative effects on both local and distant microenvironments, thereby influencing a broad spectrum of biological responses in recipient cells. These membrane-bound extracellular vesicles (EVs) play a pivotal role in delivering bioactive molecules among cells and organs. Cellular and biological processes in recipient cells, ranging from stromal cell reprogramming to immunological responses, extracellular matrix formation, and modulation of cancer cell activation, expansion, and metastasis, are subject to exosome-mediated cell-to-cell communication. Moreover, exosomes have been implicated in endowing cancer cells with resistance to treatment. Extensive research has explored the potential of exosomes as therapeutic targets and diagnostic indicators. This comprehensive review seeks to provide an in-depth understanding of the pivotal components and roles of exosomes in tumorigenesis, growth, progression, and therapeutic responses. The insights into the multifaceted involvement of exosomes in malignant cancers are essential for the scientific community, fostering the development of novel therapeutic and diagnostic strategies in the relentless pursuit of cancer.

## Introduction

Exosomes, minute lipid bilayer vesicles secreted by a majority of eukaryotic cells, have emerged as a compelling avenue for cellular waste elimination. Their role in transporting proteins, lipids, and nucleic acids from source to recipient cells underscores their pivotal function in cellular communication [1,2]. Beyond their established functions in normal physiology, heightened interest in their involvement in metabolic diseases and cancers has prompted a thorough exploration of their multifaceted properties.

Adipose tissues, among extracellular vesicles (EVs), stand out as primary producers, serving as the fundamental conduit for intercellular communication. Notably, exosomes originating from adipose tissue (AT-exosomes) have been implicated in a spectrum of physiological and pathological processes, including metabolic disorders and cancers [3,4]. The intricate involvement of AT-exosomes in cancer development, growth, and metastasis positions them as crucial components deserving in-depth scrutiny. Despite comprehensive reviews delving into the broader role of exosomes and extracellular vesicles in cancer communication [2,5–9], the exclusive focus on adipose-derived exosomes remains limited. This review centers purely on adipose-derived exosomes, elucidating their inherent functions and intricate interactions with cancer cells. The study’s tripartite structure encompasses: (a) elucidating adipocyte exosomes and their normative functions; (b) exploring the interplay between adipocyte exosomes and the tumor microenvironment; and (c) contemplating future research trajectories.

### Exosomes: overview

Extracellular vesicles encompass two major categories: microparticles, micro-vesicles, and apoptotic bodies spanning 50–1000 nm, and exosomes, extracellular lipid bilayer vesicles with diameters ranging from 30–200 nm [10]. Delving into the biogenesis of exosomes unveils a complex sequence initiated by endosomal membrane invagination and the creation of early secretory endosomes. These events culminate in the formation of late endosomal multivesicular bodies (MVBs) or endosomes, where intraluminal vesicles containing cytosolic proteins and nucleic acids are generated. Exosome cargo comprises proteins, lipids, and nucleic acids, including DNA, RNA, and microRNA, playing pivotal roles in signal transduction and genetic information exchange [1,2].

**Figure 1:**
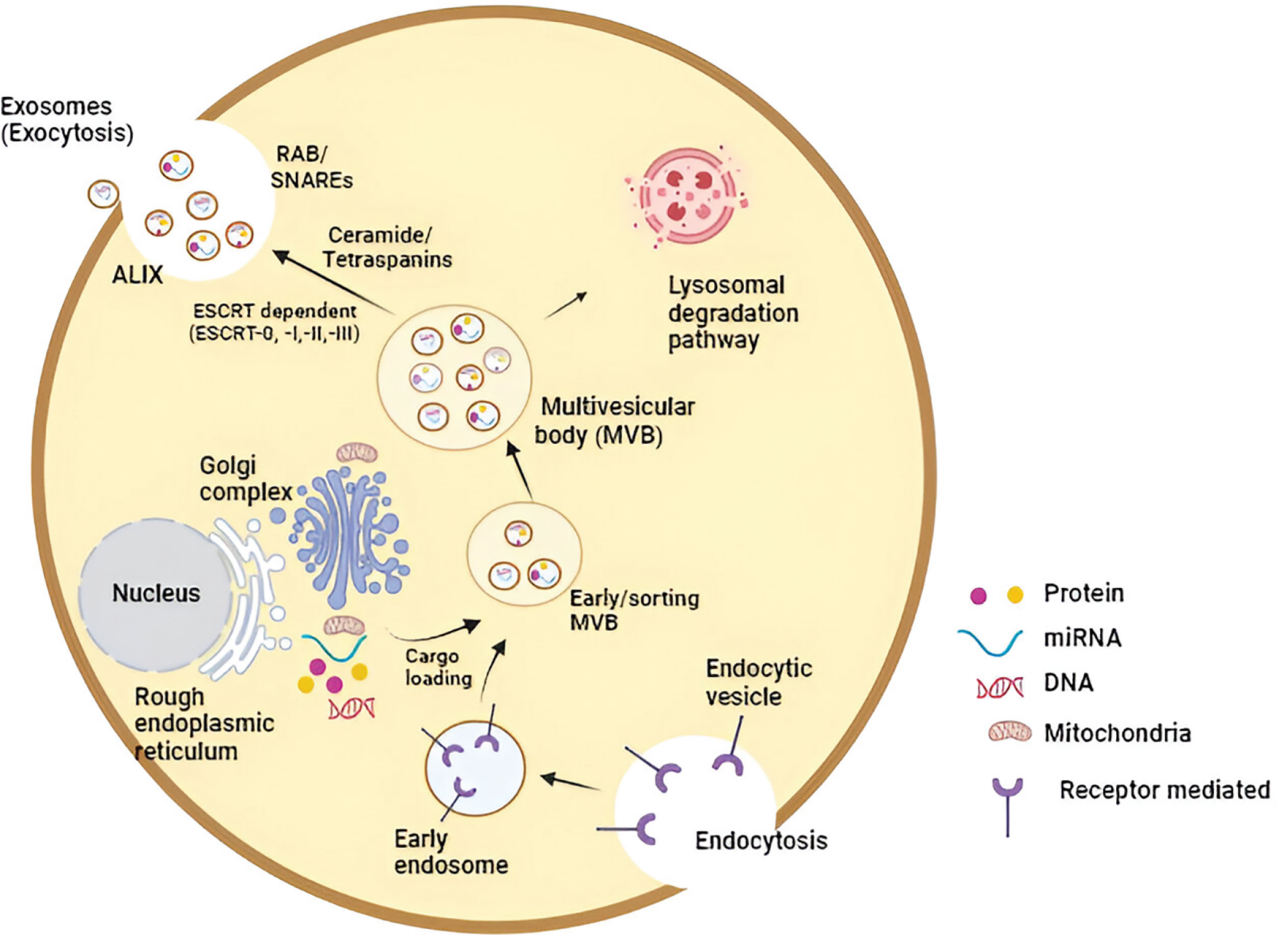
The molecular intricacies governing exosome biogenesis, inclusive of the release and uptake processes, constitute a complex cascade. In essence, the initiation of exosome biogenesis commences with the formation of an endosomal vesicle derived from the plasma membrane through the process of endocytosis. Subsequent stages involve cargo loading and sorting, culminating in the distinctive multivesicular appearance of late endosomes (MVB). The sorting of exosomal content is orchestrated by a range of mechanisms, both ESCRT-dependent and independent. Various exosomal cargoes, such as ceramides and tetraspanins, converge on the MVB membrane for loading into exosomes. The release of exosomes from MVBs into the extracellular space is facilitated either through the Rab and SNARE complex or via lysosomal degradation. These released exosomes are then available for uptake by recipient cells, achieved through either direct fusion with the plasma membrane or engagement in ligand-receptor binding (Key: miRNA—microRNA, ESCRT—endosomal-sorting complex required for transport; MVB—multivesicular body; SNARE—soluble N-ethylmaleimide-sensitive fusion attachment protein receptor; ALIX—apoptosis-linked gene 2-interacting protein X).

Research has underscored that intraluminal vesicle formation hinges on the endosomal sorting complex required for transport (ESCRT) machinery. Comprising four complexes (ESCRT-0, -I, -II, and -III), alongside key proteins such as ALIX (Apoptosis-linked gene 2-interacting protein X, encoded by PDCD6IP), VTA1 (Vesicle Trafficking 1), VPS4 (Vacuolar protein sorting-associated protein 4), and TSG101 (Tumor susceptibility gene 101 protein), ESCRT orchestrates the intricate process. Initiation of the ESCRT process involves ubiquitin-binding subunits of ESCRT-0 identifying and sequestering ubiquitinated cargo proteins on specific regions of the endosomal membrane. Bud formation and membrane deformation commence upon interaction with the ESCRT-I and -II complexes. Subsequently, ESCRT-III converges, culminating in the completion of budding processes. VPS4, a sorting protein, imparts the energy necessary for the ESCRT-III complex to detach vesicles from the MVB membrane. Additionally, the exosomal protein Alix participates in cargo selection and endosomal membrane budding. Ultimately, these vesicles merge with the donor cell’s plasma membrane and are released as “exosomes” into the extracellular milieu. Recipient cells, located in proximity or at a distance from the donor cells, can then internalize these exosomes [11–13]. The comprehensive process of exosome biogenesis is illustrated in Fig. 1.

Examining the content of exosomes reveals a diverse array of proteins, broadly categorized into distinct functional groups. Firstly, heat shock proteins (Hsp90, Hsp60, Hsp70, Hsp27) prominently feature as part of the stress response, exerting crucial roles in protein folding. Secondly, tetraspanins (CD9, CD63, CD81, CD82, CD37, CD53) contribute to cell penetration, invasion, and fusion events. Thirdly, proteins associated with multivesicular body (MVB) formation and exosome release (Alix, VPS4, TSG101) play pivotal roles. Fourthly, proteins responsible for membrane transport and fusion, including annexins and Rab proteins, constitute another vital category. Additionally, cell adhesion-related proteins, specifically integrins, lactadherin, and intercellular adhesion molecule 1 (ICAM-1), form the fifth group. Lastly, proteins participating in cytoskeletal construction, such as actins, cofilin-1, moesin, myosin, and tubulins, make up the sixth functional group [14,15].

Furthermore, exosomes harbor a rich assortment of RNA species. Notably, microRNAs (miRs), including miR-214, miR-29a, miR-1, miR-126, and miR-320, actively participate in angiogenesis, hematopoiesis, and exocytosis. Long RNA species, such as circular RNAs (circRNAs) and long non-coding RNAs (lncRNAs), constitute another layer of complexity. CircRNAs, regulating gene and microRNA expression, influence biological processes crucial in tumor cell growth, invasion, metastasis, and progression [2,16]. Long non-coding RNAs (lncRNAs), transcripts exceeding 200 nucleotides lacking significant open reading frames, contribute significantly to cell cycle regulation, epigenetic control, and cell differentiation regulation [17,18]. Noteworthy examples include the highly expressed lncRNA TUC339 in hepatocellular cancer (HCC) cell-derived exosomes [19] and the aberrantly expressed lncRNA-ATB in gastric, lung, colorectal, and hepatocellular carcinoma (HCC) reported by Li et al. [20]. Finally, lipids play a pivotal role in exosome structure and production. Phosphatidylserine (PS), phosphatidic acid, cholesterol, sphingomyelin (SM), arachidonic acid, and various fatty acids, along with prostaglandins and leukotrienes, contribute to the durability and structural rigidity of exosomes [2].

### Adipocytes and the tumor microenvironment

Adipose tissue (AT) intricately contributes to maintaining body homeostasis by storing and releasing lipids. Functioning as an endocrine organ, AT communicates both locally and peripherally, influencing immune response and metabolic control through paracrine and endocrine activities [21,22]. Comprising white adipose tissue (WAT), brown adipose tissue (BAT), and beige fat, each division possesses distinct physical and functional characteristics. WAT adipocytes, responsible for surplus fat storage, exhibit expansive capacity, while BAT and beige fat, integral to temperature regulation and energy homeostasis, maintain active metabolisms [23,24]. The proliferation of healthy adipose tissue relies on factors such as adipose progenitor recruitment, angiogenic sufficiency, proper extracellular matrix (ECM) stimulation, and inflammation control. However, an energy imbalance prompts the storage of excess energy in the form of triglycerides within adipocytes, leading to hypertrophy and hyperplasia. This shift can transform previously healthy adipocytes into cancer-associated adipocytes (CAAs) [25].

Studies, such as that by Dirat et al. [26], utilizing a two-dimensional coculture technique, demonstrated that adipocytes cocultured with murine and human tumor cells undergo phenotypic alterations resembling CAAs. These changes include de-lipidation, reduced adipocyte marker expression, and increased expression of IL-6, IL-1, and MMP-11. Co-cultivated tumor cells with mature adipocytes also exhibited heightened invasive abilities. CAAs, akin to adipocytes but larger and less vascularized, experience hypoxia, fostering extensive fibrosis, aberrant ECM remodelling, and heightened inflammation. This compromises their ability to store fat and fulfill crucial endocrine functions. Under hypoxic conditions, angiogenic agents (VEGF and FGF2) and adipokines (IL-6, TNF, IL-1, IL-8, IL-10, MCP-1, leptin, plasminogen activator 1, angiotensin II, etc.) are secreted, triggering an angiogenic response [27,28].

Chronic inflammation resulting from an enhanced immunological response facilitates macrophage recruitment and penetration, creating a pro-tumorigenic environment supportive of tumor growth. Adipocytes near malignancies undergo significant phenotypic changes, manifesting aberrant morphology and function [29]. Histologically, CAAs undergo “adipocyte dedifferentiation,” adopting fibroblast-like phenotypes with altered hormone and adipokine synthesis and release. These CAAs share characteristics with cancer-associated fibroblasts (CAFs), fibroblast-like cells implicated in cancer development and spread [30].

Additionally, adipose tissue invasion by cancer cells is noted in various cancers, including prostate, pancreatic, kidney, and colon cancers, serving as a biological predictor of tumor aggressiveness and poor prognosis [31,32]. Persistent dysregulation of proinflammatory and anti-inflammatory modulators leads to adipose tissue loss, known as cancer cachexia. Cachectic adipose tissue exhibits increased recruitment of activated macrophages, heightened gene expression of activated macrophage markers, and elevated inflammatory cytokines like IL-6 and TNF [33,34]. Enhanced triglyceride lipolysis, increased lipid mobilization, and decreased lipogenesis characterize this condition. Free fatty acids released from adjacent adipocytes are then taken up and recycled by beta-oxidation in cancer cell mitochondria, potentially fueling tumor growth by providing additional energy substrates [35]. Extracellular vesicles (EVs), released by adipose tissue, act as mediators of cell-to-cell contact and play a crucial role in the intricate interplay between adipose tissue and tumor cells. Adipocyte-derived exosomes (AdExos), a specific subtype of EVs, carry biomolecules such as miRNAs, lncRNAs, lipids, and proteins that regulate pathways involved in cancer formation and progression [36]. The subsequent section outlines the diverse ways in which exosomes from adipocytes contribute to shaping a tumor microenvironment, accompanied by relevant research findings. Notably, this review exclusively focuses on adipose-derived exosomes, excluding studies concentrated on exosomes released by cancer cells.

### Adipose-derived exosomes (AdExos) and tumor crosstalk

Numerous studies have unequivocally demonstrated the continuous interaction between adipocytes and cancer cells [37,38]. This dynamic crosstalk establishes a feedback loop wherein obesity and associated adipocytes create an environment conducive to the tumor microenvironment (TME), while tumor cells reciprocally induce malfunctioning in adipocytes. Adipose-derived exosomes (AdExos) emerge as pivotal participants in this metabolic discourse, serving as crucial mediators of cell-cell communication. They facilitate the horizontal transfer of mRNAs, short RNAs, microRNAs, proteins, enzymes, and other molecules between diverse cell types, establishing connections between afflicted cells and healthy ones.

#### AdExos in cancer cell growth and progression

The initiation of cancer involves multifaceted contributions from AdExos, which play a pivotal role in promoting tumorigenesis. Angiogenesis, the formation of new blood vessels, stands as a crucial support system for cancer cell development and growth. The adipose tissue secretome, rich in pro-angiogenic proteins, endows AdExos with a cargo abundant in pro-angiogenic factors such as IL-8, CCL2, and VEGF-D [39]. Additionally, Wang et al. [40] demonstrated that adipose tissue mesenchymal cells, when incubated with vascular endothelial growth factor, secreted exosomes rich in miR-132. This microRNA promotes lymphangiogenesis and neovascularization, as evidenced by Ma et al. [41]. AdExos carry a substantial load of miR-31, a microRNA implicated in promoting angiogenesis, inducing migration, and fostering tube-like formation in human umbilical vein endothelial cells (HUVECs) [42]. Further investigations reported that exosomes released from mouse preadipocytes (3T3L1) contained SRY-box transcription factor 9 (SOX-9). SOX-9’s pivotal role in regulating the tumor microenvironment (TME) has been established, with its activation associated with cancer development and tumorigenesis, notably by promoting angiogenesis and immune evasion of cancerous cells [43,44]. In another study, La Camera and colleagues [45] delved into the intricate role of AdExos in driving breast cancer (BC) progression and the underlying molecular mechanisms. Human MCF-7 and MDA-MB-231 BC cell lines were employed, along with fully differentiated 3T3-L1A cells to isolate adipocyte-derived extracellular vesicles (EVs). Treatment with AdExos induced a significant augmentation in the migration and invasion capabilities of MCF-7 and MDA-MB-231 cells, as substantiated by rigorous *in vitro* assays. Concurrently, exposure to AdExos resulted in a notable elevation of MMP-2 and MMP-9 levels in the extracellular media of BC cells.

Given the pivotal role of Epithelial-to-mesenchymal transition (EMT) in the genesis of cancer stem cells and the metastatic dissemination of breast cancers [46], the study probed whether AdExos could modulate the expression of critical EMT-associated genes. Among these genes, HIF-1α (Hypoxia-inducible factor 1-alpha) exhibited the most pronounced up-regulation at the mRNA level compared to control cells. HIF-1, a crucial transcription factor facilitating the adaptation of cancer cells to hypoxic microenvironments, emerged as paramount for BC invasion, migration, and metastasis [47,48]. Furthermore, the research team identified an overexpression of HIF-1 downstream target genes, including VEGF (Vascular Endothelial Growth Factor), Leptin (Ob), MMP-2, MMP-9, and Plasminogen Activator Inhibitor 1 (SERPINE1) in AdExos-treated BC cells. Importantly, suppression of HIF-1 expression in BC cells not only abrogated the activation of the VEGF promoter by AdExos but also mitigated the AdExos-induced effects on the motility and invasiveness of BC cells. The investigation extended to *in vivo* models, employing nude mice for lung metastatic colonization studies. MDA-MB-231 cells, either alone or co-administered with 3T3-L1A-EVs and KC7F2 (an inhibitor of HIF-1 activity), revealed a compelling narrative. Mice receiving AdExos-conditioned BC cells exhibited a significantly augmented metastatic load in the lungs compared to untreated controls. Notably, mice treated with KC7F2 and AdExos-conditioned BC cells demonstrated a remarkable 40% reduction in the burden of lung metastases, unequivocally implicating the involvement of the HIF-1 protein. These findings underscore the potential exploration and application of anticancer treatment modalities, particularly HIF-1 inhibitors, for the future care of BC patients, particularly those grappling with obesity.

#### Hypoxia and AdExos

Hypoxia, characterized by reduced tissue oxygen saturation, stands as a prominent feature within the Tumor Microenvironment (TME), stemming from elevated oxygen demand or increased blood volume due to excessive cell proliferation [49,50]. Prolyl-4-hydroxylases (PHDs) normally hydroxylate HIF-subunits under physiological conditions, guiding them for proteasomal degradation. However, in the presence of hypoxia, this breakdown mechanism is hindered, allowing HIF-1 subunits to translocate into the nucleus and form a heterodimeric complex with HIF-1 (HIF1B). This complex binds to hypoxia-responsive elements (HREs) in the promoter regions of downstream target genes, including vascular growth factors VEGF-A and PDGF, activating their expression [51,52]. Concurrently, signalling pathways supporting tissue survival, cell proliferation, and an intensified inflammatory response are activated, involving nuclear factor NF-kB, mTOR, and STAT3 [53,54]. In conditions of oxidative stress, an excess of reactive oxygen species (ROS) is generated. Notably, elevated plasma exosome levels in cancer patients compared to healthy individuals indicate that hypoxic conditions induce heightened exosome release from diverse cancer cells [15].

Hypoxia profoundly impacts the size, quantity, and cargo expression of exosomes [55]. While existing literature predominantly explores cancer cell-secreted exosomes under hypoxic conditions and their targeted effects on cancer progression, our discussion narrows its focus to the role of hypoxia-induced adipose-derived exosomes. Under physiological conditions, the adipose tissue microenvironment, comprising mature adipocytes and preadipocytes, maintains optimal vascularization, releasing anti-inflammatory cytokines (e.g., IL-4, IL-10, and IL-13) to uphold metabolic homeostasis [56,57]. However, advancing obesity leads to adipose tissue hyperplasia, hypertrophy, and cellular stress due to compromised vascular supply. Lipid overload and adipocyte malfunction contribute to hypoxia within adipose tissues. In a study utilizing 3T3-L1 adipocyte models, Sano et al. [58] demonstrated that a hypoxic environment enhances exosome proteins involved in lipid synthesis, including acetyl-CoA carboxylase, glucose-6-phosphate dehydrogenase, and fatty acid synthase. Under hypoxic conditions, the total number of proteins released by exosomes increased three to fourfold compared to normoxic settings.

In the context of hypoxic adipocyte-derived exosomes and their impact on nasopharyngeal carcinoma (NPC) progression, a study revealed that these exosomes, under hypoxic stress, secreted lower levels of miR-433-3p compared to normoxic exosomes. The overexpression of HIF-1 in hypoxic adipocytes was identified as a regulator, reducing miR-433-3p expression in adipocyte-derived exosomes in response to hypoxic stress. This reduced expression of miR-433-3p promoted migration, proliferation, and lipid accumulation in NPC cells [58].

#### Hypoxia-induced angiogenesis: revealing AdExos’ role

The orchestration of angiogenesis under hypoxic stress stands as a pivotal factor influencing tumor progression. Tumor strategically disturb the delicate balance between proangiogenic and antiangiogenic factors to fulfil their exigencies for growth, invasion, and metastasis development [59]. Moreover, the stromal vascular fractions of adipose tissue, when exposed to hypoxia, significantly contribute to the synthesis of proteins pivotal for angiogenesis and tissue regeneration [60]. Gangadaran et al. [39] conducted a seminal study isolating stem cells from human adipose tissue (ADSCs) and subsequently extracting extracellular vesicles, including exosomes, from these stem cells. The cargo of these exosomes revealed a repertoire of angiogenic proteins, prominently featuring interleukin 8 (IL-8), chemokine (C-C motif) ligand 2 (CCL2), tissue inhibitor of metalloproteinases 1 (TIMP-1), TIMP-2, and vascular endothelial growth factor-D (VEGF-D). *In vitro* assessments demonstrated that exosomes from adipose tissue stem cells significantly enhanced endothelial cell proliferation, migration, total vessel length, total number of junctions, and junction density. The pro-angiogenic potential of exosomal proteins was further corroborated through an *in vivo* Matrigel plug experiment.

Building on this foundation, Kang et al. [42] revealed that microvesicles secreted by adipose-derived stem cells, enriched with miR-31, substantially elevated the migration and tube formation of human umbilical vein endothelial cells (HUVECs). Moreover, pro-angiogenic exosomal miRNAs, including miR-126, miR-130a, and miR-210, exhibited significant upregulation compared to normoxic exosomes [61]. Wang et al. [40] delved into the mechanisms governing VEGF-C-dependent lymphangiogenesis promoted by exosomes derived from adipose-derived stem cells (ADSCs). Their *in vitro* experiments illustrated that ADSCs/VEGF-C not only increased lymphatic endothelial cell (LEC) proliferation, migration, and tube formation but also showcased a more robust effect than ADSC exosomes alone. Additionally, exosomes from VEGF-C-treated ADSCs displayed markedly elevated levels of miR-132 compared to control ADSC exosomes, further accentuating their role in fostering a lymphangiogenic response. Collectively, these studies underscore the integral involvement of AdExos in hypoxia-induced tumor angiogenesis, thereby fueling subsequent tumor development and metastasis. The adipose tissue secretome emerges as a crucial contributor, facilitating the release of pro-angiogenic proteins that, in turn, support the infiltration and dissemination of cancer cells.

#### AdExos-mediated metabolic reprogramming via FAO

Lazar et al. [62] presented a compelling investigation into the role of AdExos in propelling melanoma migration and aggressiveness through fatty acid oxidation (FAO)-based pathways. The significance of FAO in cancer progression, elucidated by Carracedo et al. [63], underscores its role as an additional source of essential ATP and NADPH for cancer cells’ growth and survival. Post-exosome isolation and purification from mature adipocytes, melanoma cells treated with AdExos exhibited heightened migration and invasion. Mass spectrometry analysis of the AdExos proteome unveiled a prevalent presence of FAO-related proteins (ECHA and hydroxyacyl-coenzyme A dehydrogenase), indicating AdExos’ involvement in inducing metabolic reprogramming favoring FAO in recipient cancer cells. Pharmacologic inhibition of FAO effectively reversed the impact of AdExos on cancer cell migration, emphasizing the pivotal role of FAO in this deleterious metabolic reprogramming associated with obesity. Similar observations by Xu et al. [64], linking upregulated genes related to oxidative phosphorylation and fatty acid metabolism in advanced melanoma cells, accentuate the potential relevance of FAO inhibition as an intervention strategy in cancer treatment.

#### AdExos and cancer cell proliferation via the hippo signalling pathway

The upregulation of the Hippo signaling pathway, implicated in carcinogenesis, emerges as another mechanism through which AdExos enhance cancer cell proliferation and invasion [65,66]. Wang et al. [67] isolated exosomes from *in vitro* mesenchymal stromal/stem cell (MSC)-differentiated adipocytes and introduced them to breast cancer cells (MCF7). The results showcased active absorption of these exosomes by breast cancer cells, amplifying proliferation and migration. Removal of exosomes from MSC-differentiated adipocyte-conditioned medium led to diminished proliferative and migratory abilities in MCF7 cells. Intriguingly, these exosomes protected breast cancer cells from apoptosis induced by the chemotherapy drug 5-fluorouracil (5FU) and serum starvation conditions, underscoring their role in apoptosis resistance. Transcriptome analysis highlighted the activation of Hippo signaling pathways, with downstream effector proteins YAP and/or TAZ being notably engaged. Lin et al. [66] corroborated these findings, revealing that exosomal miR-4800-3p plays a pivotal role in accelerating hepatocellular carcinoma development by modulating the Hippo pathway. While promising, further comprehensive data on AdExos’ role in cancer progression via the Hippo signaling pathway is warranted for a thorough understanding.

#### Wnt/β-catenin signaling pathway and AdExos

The Wnt/β-catenin signalling pathway, a highly conserved system crucial for tumor growth, maintenance, cell fate determination, embryonic patterning, and morphogenesis [68], takes centre stage in the study by Lin et al. [69]. Exosomes from human adipose-derived MSCs, identified and characterized, exhibited a dose-dependent enhancement of migratory and proliferative potential in MCF7 breast cancer cells following pre-treatment. Gene expression profiles and gene ontology analyses highlighted the pronounced elevation of the Wnt signaling pathway in MSC-exosome-treated MCF7 cells. Subsequently, beta-catenin mRNA and protein levels significantly increased, accompanied by the upregulation of WNT target genes Axin2 and Dkk1. This observation was further validated by Rios-Colon et al. [36], who demonstrated that pre-adipocyte-derived exosomes facilitated breast cancer cell growth *in vivo*, while MSC-differentiated adipocyte-derived exosomes accelerated tumor progression in MCF-7 cells by modulating the Wnt/β-catenin pathway.

#### AdExos facilitate cancer invasion and migration via MMP9 upregulation

Wang et al. [70] shed light on a novel mechanism underlying AdExos’ promotion of cancer cell invasion and migration. Investigating 3T3-L1 adipocyte-derived exosomes (3T3-A-EXO) on murine 3LL Lewis lung cancer cells, the study revealed enhanced *in vitro* invasion capacity in 3LL cells treated with 3T3-A-EXO. Examination of MMP2, MMP3, MMP9, and cathepsin B levels in 3T3-A-EXO-treated 3LL cells indicated a significant increase in MMP3 activity. Inhibition of MMP3 with a specific inhibitor hindered the invasive ability of 3LL cells pre-treated with exosomes. MMP3 activation, in turn, triggered MMP9 in 3LL cells, emphasizing the crucial role of MMP3, packaged into exosomes, in promoting tumor invasion. This finding aligns with previous research linking exosomes released by activated T cells to melanoma and lung cancer cell metastasis through MMP9 expression [71]. The study by Wang et al. suggested that exosome-mediated protein transfer, particularly the elevated MMP3 levels observed in obese lung cancer patients, may contribute to lung cancer metastasis in the context of obesity.

#### AdExos confer chemoresistance in cancer cells via suppression of ferroptosis

Ferroptosis, a distinctive form of programmed cell death reliant on iron, characterized by lipid peroxide and reactive oxygen species (ROS) accumulation, offers unique avenues for cancer therapy [72,73]. Zhang et al. [74] explored AdExos’ role in mediating ferroptotic chemoresistance in colorectal cancer (CRC) cells. The study spotlighted microsomal triglyceride transfer protein (MTTP), highly expressed in adipose tissue (AT), orchestrating lipid metabolism to inhibit lipid ROS peroxidation through the MTTP/PARP/Zinc finger E-box binding homeobox 1 (ZEB1) axis. Elevated MTTP expression in CRC patients with high body fat ratios, delivered by adipose-derived exosomes, resulted in ferroptosis inhibition. This led to chemoresistance against oxaliplatin through increased GPX4 and xCT expression. *In vivo*, AdExos-delivered MTTP reduced oxaliplatin sensitivity, emphasizing a promising avenue to enhance chemotherapy outcomes.

#### AdExos-secreted miRNA confers chemoresistance

miRNAs, key components within exosomes, regulate target gene expression, contributing to cancer cell communication [75,76]. Au Yueng et al. [77] isolated exosomes from ovarian cancer-associated fibroblasts (CAFs) and adipocytes (CAAs), revealing significant enrichment of microRNA-21 (miR21) isomiRNAs. Functional experiments demonstrated that exosomal miR21 from CAAs or CAFs increased ovarian cancer cell motility and invasiveness, conferring chemoresistance against paclitaxel. Exosomes transferred miR21, targeting APAF1, to ovarian cancer cells, reducing apoptosis. The study unveils the potential therapeutic strategy of blocking stromal-derived miR21 transfer to manage metastatic and recurrent ovarian cancer, highlighting a novel approach to impede chemoresistance.

#### AdExos in tumor-associated immunology

**Figure 2:**
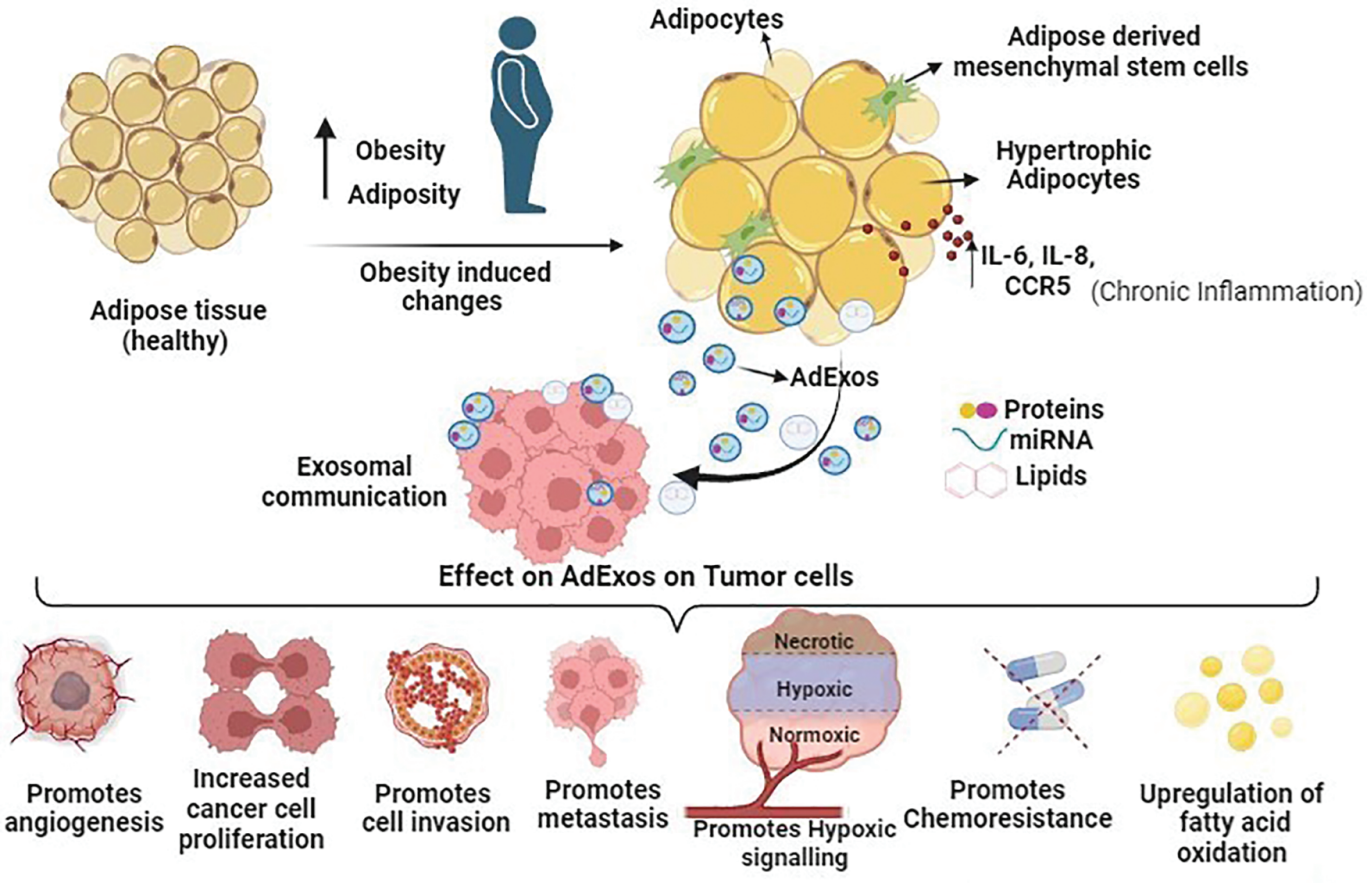
Schematic overview illustrating the impact of adipose tissue-derived exosomes (AdExos) on tumor cells, driving cancer cell growth, invasion, progression, chemoresistance, and metastasis. In summary, obesity initiates the expansion of adipose tissue, characterized by adipocyte hyperplasia, hypertrophy, and elevated cytokine-related signaling pathways (IL-6, IL-8, and CCR5 in macrophages), fostering a persistent inflammatory state conducive to the tumor microenvironment (TME). Adipose tissue releases a plethora of extracellular vesicles (EVs), among which AdExos play a crucial role in mediating communication between adipose tissue and cancer cells. The secreted components of AdExos (mRNAs, short RNAs, microRNAs, proteins, enzymes, and other molecules, etc.) significantly contribute to key cancer hallmarks, including the promotion of cancer cell growth, facilitation of cell invasion, advancement of progression, induction of chemoresistance, and orchestration of metastasis, all mediated through various pathways as detailed in this manuscript.

The immune system, encompassing T cells, neutrophils, and macrophages, traditionally defends against potential cancer cells and infections. However, obesity triggers adipose tissue expansion, marked by adipocyte hyperplasia, hypertrophy, and heightened cytokine-related signaling pathways (IL-6, IL-8, and CCR5 in macrophages), fostering a chronic inflammatory state favoring the tumor microenvironment. Consequently, inflammatory cells undergo functional shifts from immune defense to promoting tumor growth. Obesity-induced adipose tissue enlargement leads to a T cell phenotype shift towards exhaustion, marked by PD-1 upregulation [78]. PD-1 overexpression initiates inhibitory signaling, diminishing cytotoxic T cell anticancer efficacy [79]. In hepatocarcinoma and melanoma, an obesity-related animal model linked increased tumoral PD-L1 expression to reduced CD8+ and exhausted phenotypes in tumor-infiltrating lymphocytes [80]. Adipose tissue-derived exosomes contribute to M2-like macrophage polarization, fostering tumor growth [81,82]. M2-like macrophages, expressing immunosuppressive molecules like IL-10 and PD-L1, promote neovascularization and angiogenesis through adrenomedullin and VEGFs, correlating with aggressive cancer types [83–85]. Similarly, tumor-associated neutrophils (TANs) exhibit cytotoxic (N1) and immunosuppressive (N2) phenotypes. Exosomes from adipose tissue mesenchymal stem cells (ADMSCs) decrease neutrophil apoptosis, enhance phagocytic capacity, and shift towards an N1-like profile when internalized. This dual role of exosomes in modulating immune responses signifies their potential in cancer treatment [86,87]. Diagrammatically summarized in Fig. 2 are the diverse effects of adipose tissue-derived exosomes on tumor cells, impacting cancer cell growth, invasion, progression, chemoresistance, and metastasis.

## Conclusion

Herein we elucidate the pivotal role played by adipose-derived exosomes within the complex landscape of cancer progression. A robust association emerges between adipose tissue hypertrophy in the context of obesity and the intricacies of the tumor microenvironment. This connection is orchestrated through the participatory role of exosome-mediated crosstalk between cells in states of affliction and those in a healthy state. The profound correlation of AdExos with cancer hallmarks, encompassing proliferation, resistance to cell death, angiogenesis, invasion, metastasis, and immunological response, hinges upon the intricate interplay of multiple pathways expounded upon in this comprehensive article. Notably, Table 1 succinctly highlights pivotal studies in this specific domain. While recognizing the significance of AdExos in the cancer landscape, it is imperative to underscore the necessity for further studies. These should delve into exploring the utility of AdExos as potential tumor biomarkers and avenues for anticancer therapies. However, the execution of such studies encounters notable technical limitations. A significant drawback lies in the absence of standardized methods for the isolation and purification of exosomes, with conventional techniques relying heavily on multi-step ultracentrifugation. This process often yields exosome samples contaminated with various extracellular vesicle forms, introducing laborious challenges [88]. Furthermore, the characterization of exosomes grapples with the inherent heterogeneity of extracellular vesicle isolates, diverse size distributions, challenges in cargo content analysis, and issues related to microscopy [89]. Comprehensive reviews by Li et al. [89] and Hussen et al. [90] adeptly outline the challenges ingrained in exosome research while offering potential solutions. Nevertheless, given that obesity stands as a significant risk factor for the initiation and exacerbation of cancer progression, it is incumbent upon the scientific community to recognize and address these primary challenges. By staying attuned to recent developments, we can advance adipose tissue-derived exosome-based research, paving the way for medical applications that hold promise for a brighter tomorrow.

**Table 1:** Highlights from studies focusing on exploring intervention approaches using adipose-derived exosomes against cancers

Adipose derived exosome based target drug	Type of cancer	Major findings	Reference
Adipose stem cell derived exosomes secreted miR-145	Breast cancer	Overexpression of Exo-miR-145 by plasmid inhibited breast cancer cell growth in MCF-7 cells and induced cell apoptosis.	[91]
Adipose stem cell derived exosomes secreted miR-122	Hepatocellular carcinoma (HCC)	Overexpression of Exo-miR-122 was shown to inhibit cancer cell proliferation, metastasis, increase cell apoptosis and enhance the chemosensitivity of HCC cells.	[92]
Adipose tissue-mesenchymal stem cells (AMSC) derived exosome secreted miR-122	HCC	Exo-miR122 rendered cancer cells sensitive to chemotherapeutic agents and intra-tumor injection of 122-Exo significantly increased the antitumor efficacy of sorafenib on HCC *in vivo*.	[93]
AMSC derived exosome miR-320	Colon cancer	Ectopic expression of miR-320 led to inhibit HCT-116 cell proliferation, invasion and hypersensitivity to 5-Fu and Oxaliplatin involving FOXM1 as its direct target.	[94]
AMSC derived exosome miR-320	Colorectal cancer (CRC)	Lentiviral-mediated re-expression of miR-320c inhibited HCT116 CRC growth and migration *in vitro*, sensitized CRC cells to 5-Fluorouracil (5-FU), and inhibited cancer formation in SCID mice.	[95]
AMSC derived exosome miR-148b	Endometrial cancer	miR-148b suppressed cell proliferation and regulated the oxidative stress response in human endometrial cancer RL95-2 cells by inhibiting endoplasmic reticulum metalloprotease 1 (ERMP1) expression.	[96]
Adipose stem cell derived exosomes secreted miR-1236	Breast cancer	adMSC-Exos secreted miR-1236 enhanced the sensitivity of breast cancer cells to cisplatin (DDP) involving the downregulation of SLC9A1 downregulation and Wnt/β-catenin inactivation.	[97]
AMSC derived exosome secreted miR-199	HCC	AMSC-Exo-199a significantly sensitized HCC cells to doxorubicin by targeting mTOR pathway.	[98]
AdExo-miR-122-5p	Gastric cancer	Exo-miR-122-5p could hinder gastric cancer cell proliferation and metastasis *in vitro* and cancer growth *in vivo*.	[99]
Adipose stem cell derived exosomes secreted miR-138-5p	Bladder cancer	Exo-miR-138-5p prevented the migration, invasion, and proliferation of bladder cells *in vitro* and cells *in vitro* and suppressed the growth in subcutaneous xenograft mouse model as well.	[100]

## Data Availability

All the data is available in the manuscript and there is no associated data.
